# Biofortification and phytoremediation of selenium in China

**DOI:** 10.3389/fpls.2015.00136

**Published:** 2015-03-20

**Authors:** Zhilin Wu, Gary S. Bañuelos, Zhi-Qing Lin, Ying Liu, Linxi Yuan, Xuebin Yin, Miao Li

**Affiliations:** ^1^Key Laboratory of Agri-Food Safety of Anhui Province, School of Resources and Environment–School of Plant Protection, Anhui Agriculture University, Hefei, China; ^2^Advanced Lab for Selenium and Human Health-Jiangsu, Bio-Engineering Research Centre of Selenium, Suzhou Institute for Advanced Study, University of Science and Technology of China, Suzhou, China; ^3^School of Earth and Space Sciences, University of Science and Technology of China, Hefei, China; ^4^United States Department of Agriculture—Agricultural Research Service, Parlier, CA, USA; ^5^Department of Biological Sciences, Southern Illinois University Edwardsville, Edwardsville, IL, USA; ^6^Environmental Sciences Program, Southern Illinois University Edwardsville, Edwardsville, IL, USA

**Keywords:** biofortification, phytoremediation, selenium, deficiency, nutrient

## Abstract

Selenium (Se) is an essential trace element for humans and animals but at high concentrations, Se becomes toxic to organisms due to Se replacing sulfur in proteins. Selenium biofortification is an agricultural process that increases the accumulation of Se in crops, through plant breeding, genetic engineering, or use of Se fertilizers. Selenium phytoremediation is a green biotechnology to clean up Se-contaminated environments, primarily through phytoextraction and phytovolatilization. By integrating Se phytoremediation and biofortification technologies, Se-enriched plant materials harvested from Se phytoremediation can be used as Se-enriched green manures or other supplementary sources of Se for producing Se-biofortified agricultural products. Earlier studies primarily aimed at enhancing efficacy of phytoremediation and biofortification of Se based on natural variation in progenitor or identification of unique plant species. In this review, we discuss promising approaches to improve biofortification and phytoremediation of Se using knowledge acquired from model crops. We also explored the feasibility of applying biotechnologies such as inoculation of microbial strains for improving the efficiency of biofortification and phytoremediation of Se. The key research and practical challenges that remain in improving biofortification and phytoremediation of Se have been highlighted, and the future development and uses of Se-biofortified agricultural products in China has also been discussed.

## Introduction

Biofortiﬁcation is an agricultural process that increases the uptake and accumulation of specific nutrients (Rouached, 2013), e.g., selenium (Se), in agricultural food products through plant breeding, genetic engineering, and manipulation of agronomic practices. The development and uses of biofortiﬁed agricultural products have been proposed as a promising functional agricultural strategy to increase the dietary nutrient intake for humans (Banuelos and Lin, 2009; Zhao and McGrath, 2009; Zhu et al., 2009; Kieliszek and Blazejak, 2013; Borrill et al., 2014). Phytoremediation of Se is the use of plants and their associated microbes for environmental cleanup, through processes that include, phytoextraction, rhizofiltration, and phytovolatilization (LeDuc and Terry, 2005; Pilon-Smits, 2005; Robinson et al., 2009; Yasin et al., 2015b). Water and soil Se-contamination resulted from coal production and agricultural drainage has caused significant toxic impacts on aquatic wildlife, such as deformity of waterfowl in the Kesterson National Wildlife Refuge in central California. Phytoremediation is an alternative and sustainable remediation technology compared with traditional physical and chemical remediation approaches. Both Se phytoextraction and biofortiﬁcation processes are based on bioaccumulation of Se that involves plant uptake, distribution, accumulation, and transformation of Se from soil into the plant’s matrix (Zhao and McGrath, 2009; Zhu et al., 2009; Bañuelos et al., 2015). Although the goals of biofortiﬁcation and phytoremediation of Se are different, these two processes can sometimes be closely connected on enhancing the efﬁciency of Se uptake and accumulation in plants (Vamerali et al., 2014). Therefore, it is important to better understand the rhizosphere physical, chemical, and biological processes that affect soil Se bioavailability, plant uptake, distribution, and transformation of Se in the plant. Understanding and optimizing these critical processes will help to determine the success of biofortiﬁcation and phytoremediation of Se (Wang et al., 2014). In this review, we will focus on Se and use this nutrient as an example to demonstrate the processes of biofortiﬁcation and phytoremediation as a combined emerging concept for addressing the environmental and human health concerns.

## The Importance of Essential Micronutrient Selenium to Human Health

Selenium is a metalloid and commonly has four valence states in natural environment, including selenide (2–), elemental Se (0), selenite (4+), and selenate (6+). Selenium is an essential nutrient for humans and animals to form selenoproteins such as glutathione peroxidase (GPx) and thioredoxin reductases (TrxR; Barcelo and Poschenrieder, 2011; Meplan, 2011; Kaur et al., 2014). Selenoproteins play critical roles in reproduction, thyroid hormone metabolism, DNA synthesis, and protection from oxidative damage and infection (Sunde, 2012; Hatfield et al., 2014). Earlier laboratory and clinical trials showed some scientific evidence suggesting that Se might lower the risk of certain types of cancer (Yang et al., 1981; Clark et al., 1996; Reid et al., 2008; Wallace et al., 2009; Hatfield et al., 2014), but recent SELECT (the Selenium and Vitamin E Cancer Prevention Trial) studies indicated that this evidence is currently limited and not conclusive (Klein et al., 2011). More research is needed to confirm the potential relationship between Se daily intake and chemoprevention, especially for specific regions with specific sectors of the population.

The range between beneﬁcial and harmful Se concentrations is relatively narrow for humans and animals. The minimal Se concentration in livestock feed is 0.05–0.10 mg/kg dry forage, while the toxic Se concentration in animal feed is 2–5 mg/kg dry forage (Wilber, 1980; Wu et al., 1996). In humans, the World Health Organization (WHO) and USDA recommended the required human dietary intake of Se to be 55–200 μg/day for adults (Thomson, 2004; WHO, 2009). Selenium deficiency and low Se daily dietary intake can cause endemic diseases or other significant environmental health problems, such as Keshan disease (a degenerative heart disease observed in Keshan, China) and Kaschin–Beck disease (an osteoarthropathy that causes deformity of affected joints; Tan and Huang, 1991; Tan et al., 2002; Renwick et al., 2008). However, long-term exposure to high levels of Se can also lead to Se toxic effects. The common Se toxic symptoms include hair and nail loss and nervous system disorders that were previously observed in Se-rich areas such as in Enshi, Hubei, China (Li et al., 2012).

In plants, Se is not considered as an essential element. In general, concentrations of Se in plants grown in seleniferous soils are less than 25 mg/kg DW (Bell et al., 1992; Terry et al., 2000; Yasin et al., 2015b), except Se-hyperaccumulator species that accumulate over 1000 mg/kg Se in plant tissues (Ellis and Salt, 2003). In crop studies where soils were supplied with selenate, garlic (*Allium sativum*), onion (*Allium cepa*), leek (*Allium ampeloprasum*), and broccoli (*Brassica oleracea*) accumulated some Se as seleno-amino acid (selenomethyl cysteine, SeMeCys), while *Arabidopsis thaliana* and *Brassica juncea* accumulated Se primarily in the chemical form of selenite (Beilstein et al., 1991; Kahakachchi et al., 2004; Pilon-Smits and Quinn, 2010). Generally, SeMet is the most common dominant Se compound found in most grains, such as wheat, barley, and rye (Stadlober et al., 2001; Poblaciones et al., 2014). However, the Se hyperaccumulator *Stanleya pinnata* accumulated up to 90% of the total Se as MeSeCys in plant tissues (Freeman et al., 2006). For non-hyperaccumulating species, plants use S uptake and assimilation pathway to metabolize Se, since Se is chemically similar to S, and the uptake transporter and enzymes cannot distinguish between these two chemical analogs (Arvy, 1993). In addition, Se hyperaccumulator species use the same assimilation pathway from SeO_4_ to SeCys, but possess specialized or Se-specific transporters according to recent studies by Pilon-Smits and Quinn (2010). For example, the accumulation of selenate in *S. pinnata* was not inhibited by high concentrations of sulfate (Feist and Parker, 2001; Schiavon et al., 2015).

Plant-derived food products contain different amounts of Se because concentrations of Se in soil vary substantially in the natural environment. There are approximately one billion people facing with Se malnutrition in the world. For example, approximately 2/3 Chinese dietary Se intake is about 40 μg per day, which is significantly lower than the recommended Se dietary intake value of 55 μg per day according to the WHO (2009). The recommended nutritional intake (RNI) rate and upper limit (UL) of Se is 50 and 200 μg/day, respectively. For developing countries increasing the daily dietary Se intake by implementing the biofortiﬁcation strategies could substantially increase Se contents in food products. Previous studies on Se biofortification provide basic understanding on the biofortiﬁcation technology, potential health effects, and food safety regulations (White and Broadley, 2009; Zhao and McGrath, 2009; Zhu et al., 2009).

Like some other essential nutrients, Se is essential in small amounts but become toxic at high levels (Kieliszek and Blazejak, 2013; Sperotto et al., 2014). To minimize local environmental risk to wildlife, field sites with excessive Se need to be identified and remediated. In the past two decades, phytoremediation of Se has been evaluated and introduced as a successful biotechnology (Terry and Bañuelos, 2000; Barcelo and Poschenrieder, 2011). However, one of the difficulties associated with Se phytoremediation management is how to utilize/dispose of Se-contaminated plant waste materials harvested from phytoremediation sites. Different management options have been discussed by researchers regarding the disposal of plant waste materials, including landﬁll and incineration, but none of these options are considered sustainable or environmental-friendly. Generally, the plant materials harvested from phytoremediation sites can contain high concentrations of Se, which if not properly managed can be potentially toxic to waterfowl and wildlife via biomagnification. One alternative disposal option is to utilize Se-enriched plant materials for Se biofortification of agricultural products (Liu et al., 2011; Lin et al., 2014). If the Se-enriched plant material is used to amend agricultural soils, the decomposition of plant wastes will gradually release Se from the plant material, and bioavailable Se can be taken up by the crops (Bañuelos et al., 2015).

Recent developments in omics analysis and analytical technologies have led to incremental changes in research targeted on biofortification and phytoremediation of Se at molecular level (Shinmachi et al., 2010; Winkel et al., 2012; Harris et al., 2014; Schiavon et al., 2015; Visioli et al., 2015). New perspectives are emerging with the “Omics technologies” (e.g., genomics, transcriptomics, proteomics, metabolomics, nutrigenomics) and advanced analytical instruments such as micro-focused x-ray fluorescence elemental and chemical mapping and x-ray absorption near-edge structure spectroscopy, are available to further elucidate the speciation of Se in relation to specific molecular mechanisms for biofortiﬁcation and phytoremediation of Se (Bañuelos et al., 2011; Meplan, 2011; Hung et al., 2012; Winkel et al., 2012; Visioli et al., 2015). Biofortiﬁcation and phytoremediation of Se involves the changes in gene expression, protein modifications and influenced by genetic components. In particular, transcriptomics and proteomics approaches could help to further understand the phenotypic consequences of variations in Se status and unveiled Se targeted pathways for biofortiﬁcation and phytoremediation. These molecular targets and pathways could be used to unravel the effects of sulfur on biofortiﬁcation and phytoremediation of Se using non-Se hyperaccumulation plants (Barcelo and Poschenrieder, 2011). Generally, these “Omics” approaches and the recent development of analytical techniques and methods provide new perspectives to study the mechanisms for biofortiﬁcation and phytoremediation of Se and help identify and potentially develop new Se transporters to promote plant uptake and accumulation of Se.

## Use of Phytoremediation Plant Materials for Biofortification

Some plants are able to accumulate moderate amounts of Se and other trace elements in their leaves or stems (Bañuelos et al., 2009, 2015; Bañuelos and Dhillon, 2010). This plant extraction process has been applied to manage soluble Se in Se-laden soils and waters. As a result, Se-enriched materials produced from a phytoremediation field site can be further used as supplementary sources of Se to produce food or feedstuff, or functional Se biofortiﬁed agricultural products. Selenium-laden plant materials can be used as green fertilizers to increase Se concentrations in agricultural soils, or used as supplemental animal feed to increase dietary intake of Se by animals. For example, Indian mustard was used for the phytoremediation of Se-contaminated water and soil in agricultural lands of the San Joaquin Valley in Central California. After harvest, the Se-laden mustard plant materials were then used as biofortiﬁed Se supplement for animals (Bañuelos, 2006; Bañuelos et al., 2009). In this regard, by integrating phytoremediation and biofortiﬁcation processes, the chemical composition of plant materials harvested from phytoremediation should be of concern. The presence of other toxic metals (e.g., Cd, Hg, and As) in the plant materials could essentially jeopardize the use of phytoremediation plant materials for Se biofortiﬁcation. When phytoremediation plant materials are used as organic sources of Se or for specific nutrients for biofortiﬁcation, the connection between phytoremediation and biofortiﬁcation can be problematic, since the remediation soil sites are oftentimes contaminated with multi-pollutants (Vamerali et al., 2014). Thus, it is critically important to screen and select the appropriate plant species and to use toxic metal-free phytoremediation ﬁeld sites for integrating Se phytoremediation and biofortiﬁcation strategies. In general, there are two very basic requirements to meet this goal: firstly, the selected plant tissues should be edible, and secondly, the edible part of the plant should accumulate higher and safe concentrations of Se, but not other toxic metals (or chemical compounds). Phytoremediation strategies commonly attempt to select plant species that accumulate more pollutants in shoots to increase the phytoremediation efﬁciency, while biofortiﬁcation focuses on increasing a specific micronutrient content in edible plant tissues. If the biofortiﬁed materials are directly consumed to increase human nutrient dietary intake, a portion of the phytoremediation plant should be edible, such as broccoli (Bañuelos, 2002; Rodrigo et al., 2014).

Previous studies indicated that the manipulation of soil physio-chemical properties, such as soil pH, Eh, total organic carbon (TOC), and chelates, can affect the uptake and accumulation of Se and other nutrient elements by plants (Vamerali et al., 2014). In addition, some organic acids exuded by roots may play important roles in determining bioavailability of Se and other mineral nutrient elements in the soil. New research efforts have been made to integrate phytoremediation with biofortiﬁcation processes (Bhatia et al., 2013; Lin et al., 2014), but this is solely dependent on the element of concern, such as Fe, Zn, and Se. There are still many scientiﬁc questions that have not been well answered. For example, future research should investigate the feasibility of biofortiﬁcation of multiple micronutrients, such as increasing accumulation of both Se and Zn in crops or vegetables (Zhu et al., 2009). In this respect, zinc hyperaccumulator *Noccaea caerulescens* (formerly *Thlaspi caerulescens*) and Se hyperaccumulator (*S. pinnata*) may be suitable plant species for future consideration. The application of Zn- and Se-enriched plant materials as a green manures could signiﬁcantly increase the total content and bioavailability of both Zn and Se in the soil, which will enhances the accumulation of Zn and Se in the edible portion of crops (i.e., biofortification).

## Developing Se-Biofortified Agricultural Products for Human Health

The Se daily dietary intake rate varies considerably between countries/regions. With Se daily intake rates of <30 μg/d, Keshan disease has been reported in parts of China, Saudi Arabia, Czech Republic, Burundi, New Guinea, Nepal, Croatia, and Egypt (Yin and Yuan, 2012). In addition, many other countries, like India, Belgium, Brazil, UK, France, Serbia, Slovenia, Turkey, Poland, Sweden, Germany, Spain, Portugal, Denmark, Slovakia, Greece, Netherlands, Italy, China, Austria, and Ireland, were identified to have Se deficient areas because the levels of Se daily intake were below the WHO recommended amount of 55 μg/d. Korea, Australia, New Zealand, Switzerland, and Finland were identiﬁed as Se-adequate to Se-low areas because the levels of Se daily intake were in a range of 55–100 μg/d, while Japan, USA, and Canada were recognized as Se-high to Se-adequate countries with the Se daily intake of 100–200 μg/d (Yin and Yuan, 2012). In contrast, Venezuela was designated as a country with a high Se intake rate of 200–350 μg/d. If the Se daily intake is more than 550 μg/d, selenosis symptoms could be recorded, such as those observed in Enshi, China (e.g., hair and finger nail loss; Yin and Yuan, 2012). In China, the Se daily intake varied considerably from toxic levels in Enshi, to low levels of <55 μg/d in Suzhou, and to the deﬁcient levels of <11 μg/d in Keshan disease areas (Yin and Yuan, 2012).

Soil Se distribution varies signiﬁcantly in the world. More than 40 countries have limited natural Se resources, while about 80% of the world’s total Se reserves are located in Chile, the United States, Canada, China, Zambia, Zaire, Peru, Philippines, Australia, and Papua New Guinea (Liu et al., 2011). Although China is ranked the fourth in Se reserves worldwide (after Canada, the United States, and Belgium), Se-deﬁciency occurs in a geographic low-Se belt stretching from Heilongjiang Province in the northeast to Yunnan Province in the southwest, affecting 71.2% of Chinese land (Zhu et al., 2009). Therefore, Se food supplements are commonly needed for many Chinese people. In deficient areas of China, plant-based Se intake has been the primary source for humans and animals. Generally, Se-biofortified wheat, rice, and vegetables are available to provide supplemental Se (Zhu et al., 2009; Liu et al., 2011).

### Selenium Biofortification Strategy

Biofortiﬁcation is a biotechnological strategy, which aims to increase micronutrient contents, e.g., Se, in the edible parts of plants, animals, or mushrooms, via breeding, biotechnology, or application of Se fertilizers. These strategies are considered to be safe and effective in alleviating micronutrient malnutrition in many areas or countries (Nestel et al., 2006; Mayer et al., 2008; Zhao and McGrath, 2009). Generally, plant-based biofortiﬁcation is the most effective and commonly used approach, especially on staple crops, because it is a natural strategy for improving the lack of nutritional trace elements like Se in the world (White and Broadley, 2009). However, Se is not an essential micronutrient for higher plants, and it is metabolized via S-transport pathway into plant tissues (see above; Harris et al., 2014). In fact, the ability to absorb and accumulate Se varies significantly among plant species. Therefore, it is important to select specific plant species that can moderately accumulate Se in their edible parts for successful Se biofortiﬁcation. Plants selected for accumulating Se are useful as a “Se-delivery vehicle” to supplement Se in animal diet in many Se-deﬁcient areas. As a result, producing Se-biofortiﬁed meat products from animals fed Se-enriched animal feed could be another important approach for higher dietary Se intake. In addition, the excrements from the Se-fortiﬁed animals could also be used as an organic source of Se-rich fertilizers for staple crops.

### Agronomic Biofortification Strategies to Improve Se Nutrition

Agronomic biofortiﬁcation strategies are often based on application of mineral fertilizers to improve the Se bioavailability in the soil (White and Broadley, 2009; Mao et al., 2014). Agronomic Se-biofortiﬁcation strategies to increase crop Se contents by using inorganic Se fertilizers have been successfully implemented in Finland and New Zealand (Lyons et al., 2003; Hartikainen, 2005; Premarathna et al., 2012; Schiavon et al., 2013; Wang et al., 2013b). Different forms of Se supplied for biofortiﬁcation may result in different amounts and chemical forms of Se accumulated in plants (Brummell et al., 2011; Schiavon et al., 2013; Pezzarossa et al., 2014). Due to chemical similarity to sulfate, selenate can be readily absorbed by plants, and plant leaves can accumulate substantial amounts of selenate, but much less selenite or SeMet (De Souza et al., 1998; Zayed et al., 1999; Kikkert and Berkelaar, 2013). When organic acids are mixed with Se mineral fertilizers, Se can be chelated with organic compounds, which could increase plant uptake of Se and elevate the efﬁciency of Se fertilizers (Morgan et al., 2005; Lynch, 2007). The mixture of organic acids increased the efficiency of Se mineral fertilizers and resulted in a better developed and extensive root system (White and Broadley, 2005; Lynch, 2007; Kirkby and Johnston, 2008; White and Hammond, 2008). Moreover, the rhizosphere microbes and endophytic microbes may also play an important role in increasing phytoavailability of Se (Morgan et al., 2005; Lynch, 2007; Kirkby and Johnston, 2008; Duran et al., 2014; Lindblom et al., 2014). In this regard, the inoculation of soil with specific microbes might be beneficial for enhancing Se biofortification strategy for crops (Acuna et al., 2013; Duran et al., 2013, 2014; Lindblom et al., 2013a,b; Yasin et al., 2015a).

### Genetic Engineering for Se Biofortification

Biofortiﬁcation involving genetically modified organisms is based on genetic variations or transgenic technology to increase plants’ abilities to acquire the target micronutrients and accumulate them in edible parts of plants (White and Broadley, 2009). Additionally, genetic engineering techniques can increase the level of “promoter” substances, such as ascorbate (Vitamin C), β-carotene, and cysteine-rich polypeptides, which can accelerate the absorption of micronutrients in plants, and result in higher concentrations of mineral nutrients in plants. There is also genetic variation in the concentrations of mineral elements accumulated in the grains of most cereal species, whereby researchers indicated that concentrations of Fe and Zn in cereal grain may vary 1.5- to 4-folds among genotypes depending on the genetic diversity of the material tested (Cakmak, 2008; Tiwari et al., 2008). For example, the Se levels in different plants show a descending order: brassica > bean > cereal (Liu et al., 2011). Regarding transgenic approaches, the selenocysteine methyltransferase gene of *Astragalus bisulcatus* (two-grooved poison vetch) was introduced into *Arabidopsis thaliana* (Thale cress) to overexpress Se-methylselenocysteine and γ-glutamylmethylselenocysteine in shoots (Ellis et al., 2004; Sors et al., 2005a,b; Pilon-Smits and LeDuc, 2009), and resulted in an increased accumulation of Se. Others also reported that it is possible to mutagenize the Se-related genes in *Arabidopsis thaliana* to improve the efﬁciency of breeding Se-enriched crops at molecular level (Pilon-Smits and LeDuc, 2009). Genetic engineering as a supplementary technique to breeding, in combination with functional genomics gene technology could significantly contribute to future Se biofortification research (Poletti and Sautter, 2005).

## Selenium-Biofortified Agricultural Products in China

Considering that there are so many Se-deﬁcient regions in the world, it is promising to take advantage of Se-enriched plants/crops originating from Se-rich regions, e.g., in Enshi, China, as a natural and green resource of Se. One utilization option is to harvest the Se-enriched plants grown in Enshi to soils in other Se-deﬁcient areas as a source of organic Se fertilizer supporting forage crops and the application of this plant-based organic Se fertilizer can improve the Se status in the local soil, and likely result in crops enriched with Se. Carefully blending these Se-enriched plant materials as a forage blend for animals raised in Se-deﬁciency areas may result in Se-enriched meat products. Thirdly, the Se-enriched staple crops grown in Enshi can be regarded as naturally Se-biofortiﬁed products, and these Se-enriched products can be consumed by populations in Se-deﬁciency areas (Mei, 1985; Yuan et al., 2013). In this regard, local businesses in Enshi have developed various Se-enriched products, such as tea, rice, maize, herb, and drinks, which contribute to the on-going Se-biofortiﬁcation program in China (Yang et al., 2007).

### Selecting Se Accumulating Crop Plants

Selecting or breeding crop varieties with high Se-accumulation characteristics are essential for sustaining a successful Se-biofortification program (Broadley et al., 2006). For example, the black rice-Jinlong No. 1 (cultivated by Jilin Academy of Agricultural Sciences in China) was able to accumulate Se up to 6.5 μg/g DW (Yang et al., 2007; Yin and Yuan, 2012; Wang et al., 2013a). Jiangsu Academy of Agricultural Sciences cultivated another Se-enriched rice cultivar—Longqing No. 4 from Suzi No. 4 in Yunnan province (Yang et al., 2007; Yin and Yuan, 2012; Wang et al., 2013a), while Shanxi Academy of Agricultural Sciences developed a new black wheat cultivar that can accumulate 112.8% more Se than an ordinary wheat variety (Yang et al., 2007; Yin and Yuan, 2012; Wang et al., 2013a). Using these selected Se-accumulated species/cultivars can significantly increase the significance of a Se-biofortification program (Yang et al., 2007; Wang et al., 2013a).

### Foliar and Soil Application of Se Fertilizer

Foliar application of Se fertilizer is a popular practical way for producing Se-enriched foods in China (Pezzarossa et al., 2012; Boldrin et al., 2013; Wang et al., 2013a). Under optimal application conditions, Se concentrations in rice were significantly increased by 19.4% without reducing grain yields and protein/ash content (Fang et al., 2008). Chen et al. (2002) reported that, by foliar application of Se-fertilizer at a rate of 20 g Se/ha as sodium selenite and sodium selenate, the Se concentration in rice was significantly increased to 0.471 and 0.640 μg/g, respectively. Presently, Se-enriched rice is available in the market and its increased consumption can contribute to improving Se dietary intake as major staple foods in China. Tea is another popular Se-biofortiﬁed product in China. Hu et al. (2003) reported that, in addition to increased Se concentrations, the number of sprouts, yield, amino acid content, vitamin C content, as well as the sweetness and aroma of tea leaves were also significantly increased with the Se fertilizer application.

The application of soil Se fertilizers has increased the total Se and also bioavailable Se for plant uptake (Zhao et al., 2005; Broadley et al., 2010; Lavu et al., 2012, 2013; Premarathna et al., 2012; Hawrylak-Nowak, 2013; Smolen et al., 2014). Compared with natural biofortiﬁcation and foliar Se fertilizer application approaches, the soil Se fertilizers can be effective under uniform soil conditions. Earlier studies showed that soil Se fertilizers have successfully been used to enrich Se contents in a variety of agricultural products in Se-deficient regions, such as in Finland and China. However, Se fertilizers need to be reapplied annually, and farmers need to be carefully instructed on rates and the method of application. Generally, fruits and vegetables in China contain less than 3 μg/kg Se (wet weight), and rice contains less than 50 μg/kg without any application of Se (Yin and Li, 2011). The use of soil Se fertilizers can however increase the Se concentration in grains, fruits, and vegetables by several 100 times (Liu et al., 2011; Yin and Li, 2011). In recent years, the Se fertilizer application approach has been commonly used in agricultural production in some regions of China. Indeed, the novel concept of “functional agriculture” and biofortified agricultural products have been adopted by Chinese scientists and farmers, and received more acceptance and popularity (Banuelos and Lin, 2009; Zhao and Huang, 2010).

## Conclusions and Future Directions

Selenium is needed for the formation of selenoproteins, including the important GPx and TrxR. The gap between the beneficial and harmful levels of Se is, however, quite narrow. The Keshan disease has been related to Se deﬁciency, including a very low dietary Se intake of 11 μg per day in Keshan, Heilongjiang Province, China, while the loss of human hair and fingernails was observed with a daily Se intake reported as high as >2000 μg/d in Enshi, central China (Qin et al., 2013). The observations of both endemic diseases of Se-deﬁciency and selenosis from excessive Se in China are indicative of greatly uneven distribution of Se in the country. Although the Se concentrations in foods and the daily Se intake decreased significantly from 1963 to 2010 in Enshi, the present daily Se intake is still above the recommended maximum safe intake 550 μg/d (Mao et al., 2014). Moreover, the total soil Se concentration ranges from 20 to 60 mg/kg DW in Enshi, which is almost 150–500 times greater than the average Se content (0.125 mg/kg DW) in Se-deﬁcient areas and approximately 50–150 times greater than the soil Se concentration (0.40 mg/kg DW) in Se-rich areas in China. In contrast, there are about 76% countries located in Se-deﬁciency regions where the Se daily intake level is less than 55 μg/d for adults. In about 70% of China, Se-deﬁciency occurs in a geographic low-Se belt stretching from northeastern Heilongjiang Province to southwestern Yunnan Province. Therefore, developing the natural Se-biofortiﬁcation program in Enshi is a positive strategy. Then, Se-enriched plants or crops can be utilized as a source organic Se fertilizer to increase the Se contents in staple crops, or used as Se-enriched forage to support livestock in Se-deﬁcient areas. In addition, Se-enriched crops, such as rice, maize, wheat, can also be consumed by populations as a natural and safe Se-supplement in Se deﬁciency areas. Furthermore, the newly-identified Se-hyperaccumulator plant (*Cardamine hupingshanesis*; Yuan et al., 2013) can be planted in Enshi to yield high-Se plant materials to obtain an additional source of organic Se fertilizer for Se-biofortification practices.

## Author Contributions

ZW, LY and ML prepared the draft manuscript. GB and ZL revised the manuscript. All authors have read and provided input or assistance to the submission of the final manuscript.

### Conflict of Interest Statement

The authors declare that the research was conducted in the absence of any commercial or financial relationships that could be construed as a potential conflict of interest.
